# Modulation of Metabolic Hormone Signaling via a Circadian Hormone and Biogenic Amine in *Drosophila melanogaster*

**DOI:** 10.3390/ijms23084266

**Published:** 2022-04-12

**Authors:** Jason T. Braco, Jonathan M. Nelson, Cecil J. Saunders, Erik C. Johnson

**Affiliations:** 1 Department of Biology, Wake Forest University, Winston-Salem, NC 27109, USA; jasonbraco@gmail.com (J.T.B.); nelsjm17@wfu.edu (J.M.N.); saundecj@wfu.edu (C.J.S.); 2 Center of Molecular Communication and Cell Signaling, Wake Forest University, Winston-Salem, NC 27109, USA

**Keywords:** metabolism, adipokinetic hormone, pigment dispersing factor, dopamine, ecdysone, signaling

## Abstract

In insects, adipokinetic hormone is the primary hormone responsible for the mobilization of stored energy. While a growing body of evidence has solidified the role of adipokinetic hormone (AKH) in modulating the physiological and behavioral responses to metabolic stress, little is known about the upstream endocrine circuit that directly regulates AKH release. We evaluated the AKH-producing cell (APC) transcriptome to identify potential regulatory elements controlling APC activity and found that a number of receptors showed consistent expression levels, including all known dopamine receptors and the pigment dispersing factor receptor (PDFR). We tested the consequences of targeted genetic knockdown and found that APC limited expression of RNAi elements corresponding to each dopamine receptor and caused a significant reduction in survival under starvation. In contrast, PDFR knockdown significantly extended lifespan under starvation, whereas expression of a tethered PDF in APCs resulted in significantly shorter lifespans. These manipulations caused various changes in locomotor activity under starvation. We used live-cell imaging to evaluate the acute effects of the ligands for these receptors on APC activation. Dopamine application led to a transient increase in intracellular calcium in a trehalose-dependent manner. Furthermore, coapplication of dopamine and ecdysone led to a complete loss of this response, suggesting that these two hormones act antagonistically. We also found that PDF application led to an increase in cAMP in APCs and that this response was dependent on expression of the PDFR in APCs. Together, these results suggest a complex circuit in which multiple hormones act on APCs to modulate metabolic state.

## 1. Introduction

Throughout the Metazoa, metabolic homeostasis is governed by a number of different hormones that coordinate storage and release of energy. In *Drosophila*, insulin-like peptides (DILPS) and adipokinetic hormone (AKH) modulate energy storage and release, respectively [1,2]. The cells that express these hormones represent important points of integration that couple with changes in metabolic allocation towards different behaviors and/or physiologies (e.g., reproduction or growth). While many studies have focused on the regulation of the DILPS [3,4,5], there has been comparatively little research identifying elements that regulate the synthesis, release, and signaling of AKH.

Genetic ablation of AKH-producing cells (APCs) [6,7], blockade of AKH hormone release [7], and the loss of AKH or the AKH receptor gene (AKHR) [8] all produce consistent metabolic and behavioral phenotypes. Specifically, these manipulations cause an increase in triglyceride content and reduce levels of circulating trehalose [6,9]. The AKHR, which binds to AKH with high affinity, is expressed at high levels in the fat body, and genetic knockdown of AKHR in the fat body produces the same metabolic phenotypes associated with the loss of AKH [10,11]. In animals, a common behavioral response to nutrient depletion is increased locomotion [12,13]. This may seem counterintuitive, as hyperactivity during nutrient limitation would exacerbate energy depletion. However, it is hypothesized that starvation-induced activity is an adaptive trait that facilitates foraging [14]. Interestingly, animals lacking APCs show no changes in locomotor activity under starvation conditions [6]. Consequently, these animals have increased lifespan under starvation, presumably caused by the lack of starvation-induced hyperactivity and elevated levels of energy stores [6].

Despite our growing understanding of AKH signaling, little is known about the factors that govern AKH release. Currently, only two mechanisms are known that rely on the detection of changes in intracellular energy. AKH cells express two energy sensors: K^+^_ATP_ channels and AMPK [1,7,15]. These molecules couple to changes in cellular ATP with electrical excitability and AKH release [16]. While these two molecules are critical factors modulating AKH release, they are unlikely to be the sole mechanisms. Recent work has shown that the loss of AMPK only partially phenocopies the loss of AKH, suggesting alternative or additional mechanisms controlling AKH secretion [7].

Studies in other insects have implicated a number of diverse hormones modulating AKH release. Crustacean cardioacceleratory peptide (CCAP), tackykinin (TK), and octopamine are positive regulators of AKH release, and FLRFamide and FMRFamide act to inhibit AKH release in the locust [17,18,19,20]. In general, these experiments examined AKH titer in entire CNS preparations or whole organism in response to hormone application, thus making it impossible to assert whether these hormones acted directly to alter AKH release or if they acted through some intermediate [17,18].

Hormones such as allatostatin-A [21] and short NPF [22] provide regulatory input to APCs, as well as to insulin-like producing neuroendocrine cells [21,23]. The fact that these cell populations are provided input by both metabolic control centers indicates that they communicate nutritional status. The central neurons that express sNPF, like APCs, possess intrinsic nutrient sensors, and this property appears in functionally homologous peptides [24,25]. Therefore, it is likely that there are several neuroendocrine circuits through which APCs are informed of nutrient status [26]. This suggests a complex endocrine circuit that regulates metabolic centers.

Here, we report the identification of the complement of peptide and amine receptors expressed in the AKH cell lineage by employing single-cell RNA sequencing methods. We further assessed the functional contributions of the PDF receptor and all known dopamine receptors with specific regard to modulation of AKH cell physiology. Genetic knockdown of these receptors in AKH cells caused changes in starvation survival and locomotor activity. We further validated these results by directly testing for changes in AKH cell physiology using live-cell imaging of explanted AKH cells. We found that these hormones evoked specific changes in fluorescent reporters in AKH cells. These results not only implicate novel functions for some of these hormones but provide a better understanding of how multiple signals converge on AKH cells to modulate changes in organismal physiology.

## 2. Results

### 2.1. AKH Cells Express Multiple Receptor Molecules

To gain insight into potential regulatory elements governing AKH release, we evaluated the transcriptome of AKH cells. Individual AKH cells were identified by introducing a GFP reporter prior to microdissection and RNA sequencing. The transcriptome was assessed from five replicate individuals each that had experienced 24 h of starvation and compared with animals that were fed ad libitum. An initial analysis of the RNA sequencing of 13 billion nucleotides corresponding to 11,456 transcripts showed no significant effects of starvation on the expression levels of G protein-coupled receptor encoding genes. Consequently, all ten replicates were pooled for further analysis.

**Figure 1:**
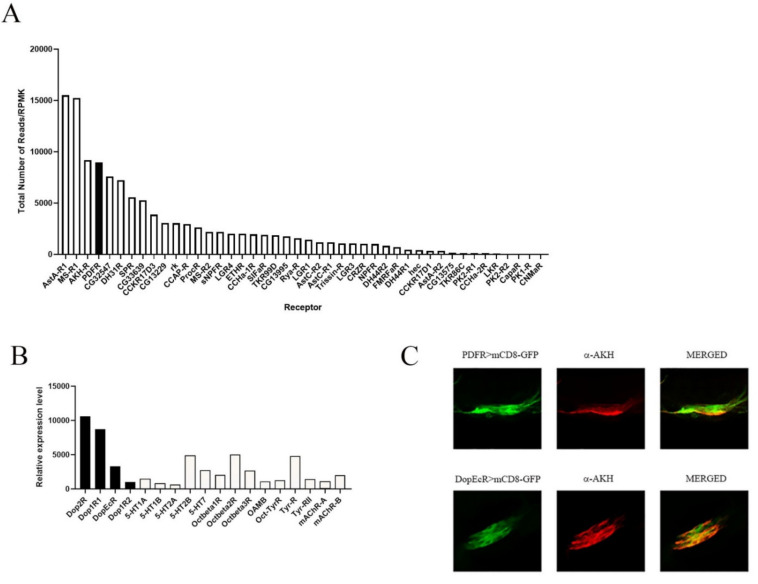
AKH-producing cells express multiple GPCRs. Results from the RNA sequencing of individual APCs were mined for specific expression of neuropeptide GPCRs (**A**) and amine GPCRs (**B**). Each gene listed was reliably expressed across samples and the reads per million mapped reads are shown here for the expressed genes. Note that the PDFR (solid bar) was the fourth most abundant receptor expressed in APCs, following the Ast-A receptor, the MS receptor, and the AKH receptor. In 1B, we note that APCs express multiple receptors for most of the small molecule transmitters, including octopamine, dopamine (designated with solid bars) and serotonin. We validated expression for the PDFR in APCs by employing a specific GAL4 element that had previously been shown to rescue PDFR-phenotypes to drive GFP and, using an antibody against AKH, showed colabeling in adult APCs (**C top**). We used a similar approach for validating the DopEcR, using a DopEcR-Gal4 element to drive GFP and counterstaining with AKH antisera (**C bottom**).

The experimental rationale for performing the RNAseq was to identify transcriptional mechanisms that regulate AKH cell physiology. We mined the AKH cell-specific transcriptome to identify which cohort of neuropeptide receptors and small molecule receptors was expressed specifically in AKH cells to offer insight into potential endocrine connections that regulate AKH cell physiology, and we considered these two classes of receptor molecules separately. As expected, the expression profile of the entire complement of neuropeptide and small molecular receptor molecules was unchanged by nutrient conditions. This was consistent with models in which GPCR regulation occurs primarily through internalization and desensitization [27]. Such modulation can occur via the interaction of the receptors with proteins such as β-arrestin [28]. Alternatively, classes of proteins may directly modify the activity and localization through posttranslational modifications of the GPCR (RAMPS and RGS) [29,30]. Consequently, we pooled the sequence reads across treatment to determine relative abundance of receptor expression. We found strong expression (defined as counts in the top third of all genes) for 13 neuropeptide receptor genes and moderate expression (defined as counts in the top two thirds of all genes) for an additional 12 receptor genes (Figure 1A). Unsurprisingly, the most abundant neuropeptide receptor in AKH cells was the AstA-R1 receptor (Figure 1A), which had previously been reported to be an upstream regulator of AKH release [21]. Likewise, previous publications had implicated the peptide myosuppressin as an inhibitor of AKH secretion in different insects [20], as well as the sNPF receptor [22]. Thus, our analysis of the transcriptome showed consistency with previous behavioral results. Therefore, we focused on the relative high expression levels of the receptor that is specifically activated by the circadian hormone, PDF, as connections between the circadian system and metabolism have been recognized in multiple taxa, but a connection between these two hormones have not yet been described.

In a set of complementary analyses, we assessed the specific expression of G protein-coupled receptors that specifically bind small molecule transmitters. Interestingly, we found expression for nearly all the classical neurotransmitters (5HT, octopamine, dopamine, acetylcholine, and tyramine) (Figure 1B). AKH cells are known to modulate octopaminergic cells and vice versa [31,32], so our transcriptome verified previous experiments on AKH cell modulation. We chose to focus on dopamine receptor expression and test for potential modulation of AKH cell physiology by dopamine. 

We first wanted to independently confirm that these molecules were expressed in AKH cells. To this end, we employed two different methods. First, we used single-cell RT PCR on dissected AKH cells and detected amplicons that corresponded to PDFR. Additionally, a PDFR driver element that has been shown to rescue relevant PDFR phenotypes [33] was used to introduce a GFP reporter to PDFR-expressing tissues. We used an AKH specific antibody [18] and found colocalization of the GFP reporter with the immunolabels (Figure 1C). Likewise, the DopEcR-GAL4 also showed strong expression in AKH cells.

### 2.2. PDFR Function in AKH Cells Is Required for Normal Behavioral Starvation Responses

**Figure 2:**
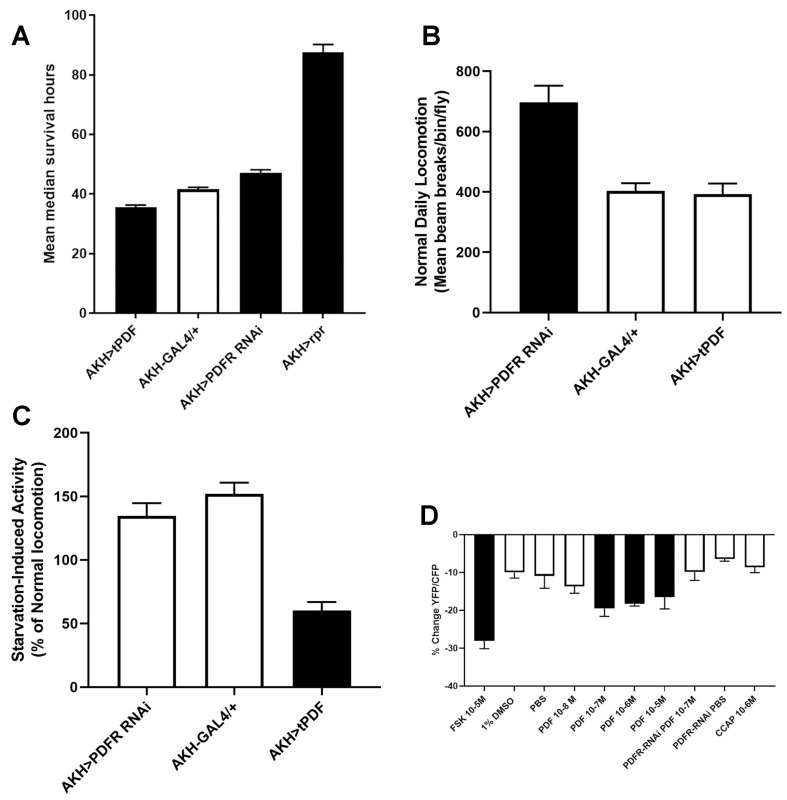
Manipulations of PDFR resulted in alterations of AKH-dependent phenotypes. We genetically manipulated PDFR function in APCs and evaluated AKH-related phenotypes, including starvation lifespan (**A**) and locomotor activity (**B**,**C**). (**A**). Specifically, APC introduction of a PDFR-RNAi element led to significantly longer mean lifespan during starvation, while introduction of a membrane-tethered PDF (t-PDF) to elicit constitutive PDFR signaling produced the opposite phenotype, i.e., a significant shorter mean lifespan during starvation. Solid bars denote significant difference from controls (*p* < 0.05 ANOVA, Tukey’s post hoc test). (**B**). Comparisons of normal locomotor activity during ad libitum conditions across these genotypes. Solid bars denote statistical significance from control lines (*p* < 0.05 ANOVA, Tukey’s post hoc analysis). (**C**). Comparisons of starvation-induced activity among different genotypes. Solid bars denote statistical significance from control lines (*p* < 0.05 ANOVA, Tukey’s post hoc analysis). (**D**). Exogenous application of PDF altered the FRET signature of the epac-camps cAMP reporter. AKH cells expressing the epac-camps FRET sensor were isolated, and different concentrations of PDF were applied. PDF elicited a significant change in FRET, consistently with previous demonstrations of this receptor modulating cAMP levels, and showed dose dependence. Notably, coexpression of a PDFR-RNAi element eliminated these responses. Solid bars denote significant differences (*p* < 0.05) from vehicle addition. Responses were generated from five replicate experiments.

After validating that AKH cells express the PDFR, we next asked whether altered PDFR expression would lead to changes in starvation sensitivity, as altered physiology of AKH cells causes a variety of starvation phenotypes [6,9]. Multiple investigations have consistently found that the loss of AKH signaling leads to increased energy stores and decreased locomotion under starvation, which together result in increased survival under starvation [6,7,9]. We hypothesized that if the PDF receptor functioned to modulate the release of AKH, then the loss of the receptor would lead to a change in lifespan under starvation. Using this experimental framework, we tested the AKH cell-specific introduction of an RNAi element targeting PDFR for altered starvation behaviors. We found that PDFR knockdown lengthened starvation lifespan (Figure 2A), suggesting that PDFR facilitates AKH release. One interesting feature of PDFR is that it does not show desensitization in response to ligand binding, and thus, persistent presentation of PDF leads to chronic activation [34,35,36]. A genetically encoded membrane-tethered PDF (tPDF) has been useful to investigate PDF gain-of-function phenotypes and does so in only cells that endogenously express the PDFR [34]. We found that expression of tPDF in AKH cells resulted in a short-lived phenotype (Figure 2A). Since PDF is a known regulator of locomotor rhythmicity [36], and AKH cells are critical for starvation-induced hyperactivity [6,9], we asked whether there were any changes in the locomotor profiles of these manipulations. PDFR-RNAi knockdown resulted in elevated activity under replete (ad libitum) conditions (Figure 2B) and a wild-type response to starvation (Figure 2C). In contrast, expression of tPDF resulted in an abnormal locomotor pattern under starved conditions (Figure 2C), with especially high relative amounts of nighttime activity (Supplementary Figure S1). These results corroborated the hypothesis that PDF signaling likely modulates AKH release. We next directly tested that hypothesis by assessing functional PDF signaling in AKH cells. The PDFR has been shown to signal predominantly through the cAMP second messenger system [37], and endogenous PDFR activation has been visualized using the epac-camps reporter [38]. Therefore, we introduced this genetically encoded cAMP reporter to AKH cells and assessed whether AKH cells were responsive to the neuropeptide PDF. We found that direct application of PDF to explanted AKH cells decreased FRET signatures of the reporter, which was consistent with elevated levels of cAMP [36]. These values were similar to the FRET changes observed with application of forskolin, a positive control that elevates cAMP levels. Notably, there was a dose dependence of PDF on changes in FRET levels (Figure 2D). In order to test the directness of this response, we repeated these experiments with a coexpressed PDFR-RNAi element. Knockdown of PDFR in AKH cells abolished PDF-induced responses, showing that PDF directly acted on AKH cells and was dependent on the specific expression of the PDF receptor.

### 2.3. Dopamine Receptor Knockdown in AKH Cells Exhibited Starvation Phenotypes

**Figure 3:**
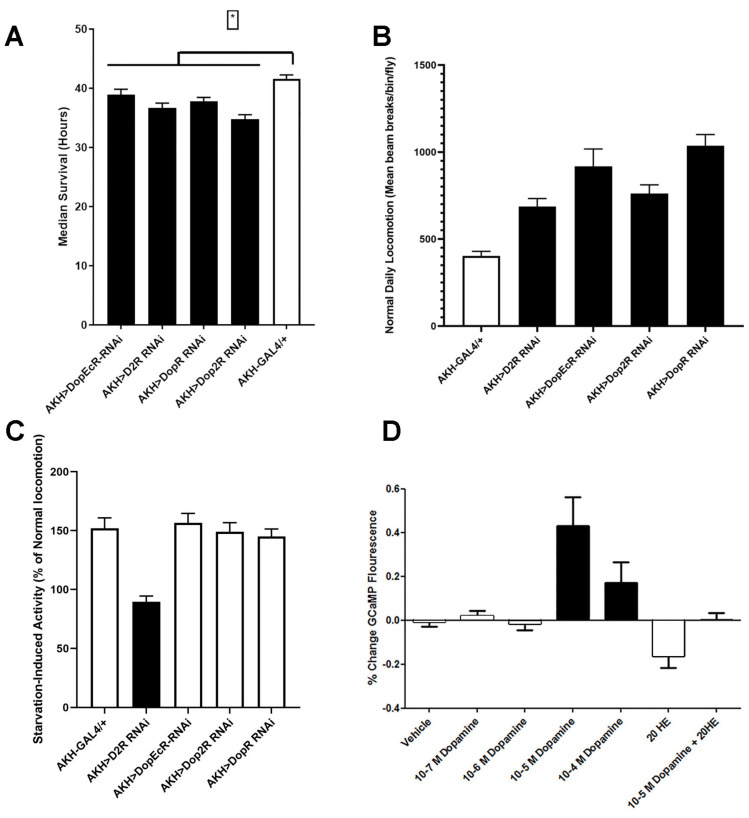
Dopamine modulated AKH-dependent phenotypes. We introduced RNAi elements targeting each of the dopamine receptors present in AKH cells and assessed starvation lifespan (**A**) and locomotor activity (**B**,**C**). Each of the dopamine receptor RNAi elements significantly reduced lifespan during starvation. Black bars denote significance (*p* < 0.05, ANOVA, Tukey’s post hoc test). (**B**). Comparisons of locomotor activity during ad libitum conditions across these genotypes, Black bars denote statistical significance from control lines (*p* < 0.05 ANOVA, Tukey’s post hoc test). (**C**). Starvation-induced activity levels in animals expressing RNAi constructs targeting dopamine receptors. Black bars denote statistical significance from control lines (*p* < 0.05 ANOVA, Tukey’s post hoc test) (**D**). Exogenous application of dopamine altered GCaMP reporter fluorescence. AKH cells expressing the GCaMP sensor were isolated, and different concentrations of DA were applied. DA elicited a significant change in GCaMP and showed dose dependence.

Dopamine is a biogenic amine that mediates a number of different behaviors and physiologies in *Drosophila* [39]. We thought it was an interesting observation that all known dopamine receptors were expressed in AKH cells. We tested for specific AKH phenotypes (starvation and locomotor activity) in animals expressing RNAi elements specifically targeting the different dopamine receptors. We found that genetic knockdown of all four individual dopamine receptors in APCs led to a significant reduction in lifespan under starvation (Figure 3A). Changes in lifespan were accompanied by aberrant locomotor phenotypes. We found that manipulations in three of the four dopamine receptors (DopEcR, Dop2R, and DopR) resulted in advanced onset of hyperactivity under starvation (Supplementary Figure S2) and overall increased locomotion during ad libitum conditions (Figure 3B), whereas conversely, D2R knockdown led to a significant decrease in starvation activity (Figure 3C). Together, these results suggest that dopamine may modulate AKH secretion. However, our results implied the involvement of multiple dopamine receptors. One of these dopamine receptors, DopEcR, has been shown to be a receptor for the steroid hormone, ecdysone, in addition to dopamine [40]. To more fully explore the potential interaction of dopamine and ecdysone in AKH cells and to gauge the contributions of multiple dopamine receptors, we monitored explanted AKH cells expressing a calcium-sensitive GFP, GCaMP6s1, in response to hormone application.

Interestingly, we found that dopamine-induced responses were dependent on the extracellular concentration of sugar. Under high extracellular trehalose levels (15 mM), dopamine application failed to produce a response. However, under low extracellular trehalose levels (3 mM), dopamine induced a strong peak in calcium in a dose-dependent manner (Figure 3D). We hypothesize that this context dependence likely reflected changes in the basal receptivity of AKH cells. We, and others, have previously reported that low extracellular trehalose concentration results in AKH cell activation; however, this was over a much longer time course (30 min), whereas peak dopamine responses occurred within 30 s after application. We next tested whether ecdysone was capable of changing calcium concentrations and found that application of 20E (20-hydroxyecdysone) resulted in no change in AKH cell activation. However, coapplication of 20-hydroxyecdysone and dopamine completely blocked any increase in calcium, suggesting that these two hormones act antagonistically in vivo (Figure 3D). Notably, coapplication of 20-hydroxyecdysone eliminated these responses.

## 3. Discussion

In this study, we identified and characterized three hormones, dopamine, ecdysone, and PDF, that are likely to directly regulate AKH secretion. Our initial exploration of the AKH cell transcriptome revealed the expression of a number of potential candidate GPCRs. We validated the expression of these receptors using multiple genetic and molecular methods. Next, we tested the behavioral consequences of knocking down these receptors in AKH cells and found that knockdown of each dopamine receptor produced aberrant changes in locomotion. Specifically, we found that three of the four dopamine receptors showed increased baseline locomotion. While the timing of hyperactivity was not investigated in detail, it appeared that aberrant dopamine signaling disrupts the onset of starvation-induced hyperactivity. These manipulations also manifested in short-lived phenotypes under starvation. We also found that changes in PDF signaling resulted in changes in lifespan and locomotion. We then functionally characterized their roles in AKH cells using live-cell imaging. Collectively, these results clearly showed that AKH cells are a critical point of integration lying at the crossroads between the physiology/behavior and metabolic state of the organism.

Our experiments identified pigment dispersing factor, PDF, as a candidate hormone regulating AKH cell physiology. PDF is best known for its paramount role in regulating circadian rhythmicity [34,36,41,42]. We postulated that PDF may act to regulate AKH cells in a circadian fashion, as recent studies have established connections between the circadian system and metabolic control [43]. The current model of PDF action is that of a wake- promoting hormone secreted in the early morning and peaking in concentration before dawn [44]. Based on the evidence of the PDF receptor (PDFR) being expressed in AKH cells, we speculated that PDF may target AKH cells to coordinate energy release in anticipation of morning activity. Introduction of an RNAi element targeting PDFR significantly extended lifespan under starvation, and in contrast, the introduction of the tethered PDF (to constitutively stimulate PDFR) decreased starvation longevity. These observations were consistent with a model in which PDF modulates AKH release.

Since PDF is a responsible for the temporal regulation of locomotor activity, we next analyzed the locomotor activity under both replete and starvation conditions. Unsurprisingly, we found that PDFR knockdown led to elevated locomotion under replete conditions and in contrast, expression of tPDF led to significantly lower activity during starvation. Considering that AKH is a requirement for starvation-induced hyperactivity, and that it appears that AKH gain-of-function variants enhance locomotor activity [9], these results appear to contradict the interpretation that PDF acts to increase AKH titers. However, comparisons of these manipulations to other AKH loss-of-function variants may be difficult. Changes in the machinery that controls AKH excitability produced changes in locomotor behavior during replete conditions, which was consistent with its role as a modulatory element [15]. Nonetheless, deviations in locomotor behavior in PDF manipulation likely indicate a functional relevance of PDF signaling on APC physiology and constituted our initial rationale for investigating this behavior. Furthermore, exogenous PDF application to explanted AKH cells resulted in increased levels of cAMP, the second messenger downstream of PDFR, and that such responses were PDFR dependent. Collectively, these results verified that PDFR is expressed in AKH cells and that PDFR action likely regulates AKH release.

Our work, as well as previous reports, suggested that AKH is dispensable for rhythmic locomotor response [6]. Although we cannot fully rule out the potential for PDF to modulate AKH in a circadian manner, the absence of any circadian phenotype in locomotion suggests that PDF may act on AKH cells in a clock-independent fashion. A major hypothesis on PDF function is that it acts to synchronize other temporal centers [42,45]. However, investigators identified a group of nonclock cells that regulate rhythmic feeding via PDF modulation [46], and other investigations have suggested that PDF may have nonclock functions. Specifically, manipulations of PDF signaling altered triglyceride levels independently of clock function [47]. Given that the circadian loci of PDF action have been mapped to a group of central neurons [38], it seems unlikely that central PDF neurons impact AKH cells. In addition to central expression, PDF is also expressed in a subset of neurons in the ventral nerve cord that project posteriorly to the midgut [48]. These PDF neurons do not express any clock genes and are dispensable for circadian rhythmicity. Furthermore, they have been shown to impact ureter contractions in the midgut and osmotic homeostasis [48]. It is possible that these PDF neurons may provide the hormone source that is upstream of AKH. PDFR is also known to respond to another ligand, DH31, albeit with comparatively lower affinity [37,49]. DH31 is a multifunctional hormone that has been linked to stress response behaviors as well as circadian rhythms [50]. Unlike PDF, DH31 is expressed in many tissues, including enteroendocrine cells in the gut [51], and therefore may be more abundant in circulating hemolymph than PDF. Consequently, it may be that DH31 signaling via PDFR is biologically relevant to AKH cells. The DH31 receptor [29] is also present in the APC-specific transcriptome, so we did not attempt to test the DH31 responsiveness of APCs. While the precise mechanism for PDFR activation in AKH cells remains unclear and is likely to be the subject of future studies, our results firmly cemented that PDFR modulates this metabolic center.

Previous literature has found that dopamine is involved in a number of different behaviors and physiologies, including courtship, memory formation, and circadian rhythms [52,53,54,55]. Interestingly, functional characterization of dopamine signaling illustrates numerous parallels with AKH signaling. Specifically, pharmacological and genetic manipulations intended to elevate dopamine signaling cause increased locomotor activity and enhanced gustatory perception of sugar [56,57,58]. Dopamine levels also rise in response to multiple forms of stress including oxidative, heat shock, and starvation [59].

These experiments also implicated ecdysone signaling as an important endocrine factor that regulates metabolic status. Both reduction in the expression of DopEcR levels and the complete loss of the receptor led to changes in locomotion levels and starvation sensitivity. The DopEcR is an interesting GPCR, as it also binds the major steroid in insects, ecdysone [40]. Specifically, ecdysone has been shown to abolish dopamine-induced responses [40] and is thought to change the signaling parameters of the receptor [60]. There are other ecdysone receptors, but we did not investigate them here for any potential functional contributions. It is worth noting that since the Ca^2+^ changes were not tested as being DopEcR-dependent, it cannot be ruled out that EcR was involved in the APC response. While ecdysone signaling is well known for its impact on mediating developmental transitions, studies have shown ecdysone signaling to be a critical mediator of stress responses in adult insects [61,62]. However, it is unlikely that EcR mediates nongenomic rapid responses such as the increase in intracellular calcium that we observed.

Like those of dopamine and AKH, ecdysone titers increase under metabolic stress [59]. Furthermore, increased ecdysone halts oogenesis in females and consequently shifts nutrient allocation from reproduction to survival [62]. From this evidence, we hypothesized that ecdysone may abolish the inhibitory effects of dopamine on AKH cells and directly tested for interactions of these two hormones. We found that administration of dopamine under high extracellular trehalose failed to induce any change in GCaMP fluorescence. However, we did find that when extracellular trehalose was low, dopamine induced a strong increase in fluorescence. Furthermore, coapplication of ecdysone eliminated any dopamine response under these nutrient levels. Ecdysone is thought to act by directly modulating DopEcR activity; in our experiment, the entirety of the dopamine-induced response was abolished by ecdysone even though other dopamine receptors are present in AKH cells. One potential hypothesis is that the other dopamine receptors signal through different mechanisms such that the calcium influx is primarily downstream of the DopEcR. Consistently with that hypothesis, the DopEcR was the most abundantly expressed dopamine receptor according to our transcriptome dataset. Furthermore, it has been previously suggested that ecdysone does not inhibit dopamine signaling per se but rather changes the second messenger that is downstream of receptor activation [60]. Our observations would be consistent with that model of DopEcR activation. One other potential mechanism that could explain these results is that the other dopamine receptors may act to modulate DopEcR signaling to precisely regulate AKH cell responsiveness to these different ligands (dopamine or ecdysone). Similarly, expression of DopEcR in Gr5a-expressing gustatory neurons is required for starvation-induced sugar sensitivity in adult flies. It could be that DopEcR functions in a similar manner in these sugar-sensing neurons as we reported in APCs. Future experiments aimed at identifying the roles of these molecules are required, as our results clearly demonstrated that dopamine and ecdysone act antagonistically on AKH cells.

While all of our independent experiments firmly established a role for dopamine and ecdysone impacting AKH signaling, how do we reconcile the different results suggesting that dopamine may inhibit or enhance AKH signaling? One hypothesis is that dopamine may act as a neuromodulator, and the idea that dopamine must either be an excitatory or an inhibitory input is likely too simplistic. This idea is supported by the observations that multiple dopamine receptor subtypes are present in AKH cells and that the dopamine responsiveness of AKH cells is dependent on extracellular sugar levels. Furthermore, interpretation of our behavioral experiments centering on animals expressing lifelong genetic constructs may not be readily comparable to interpretations of dopamine action on AKH cell physiology observed along much shorter timescales. Given the contextual dependence of dopamine signaling, we submit that loss of dopamine receptor signaling phenotypes may not simply distill into a model in which dopamine inhibits AKH secretion. Further complicating our understanding of the behavioral phenotypes is the fact that both dopamine and ecdysone rise in response to stress in vivo. Our results suggested that these two hormones act antagonistically to regulate AKH, indicating that the pertinent information is in the ratio of these two hormones. Consequently, the net contribution of this receptor to AKH cells is reliant on the precise stoichiometry of these two molecules at a given time. Furthermore, the temporal dynamics of both dopamine and ecdysone increases are unclear, and it may be that the relevant information is during stress, when these hormones are released, as well as absolute abundance. Similar limitations may apply to interpretations of PDF actions on APCs as well.

These results demonstrated that AKH cells are regulated by multiple hormones. While the predicted phenotypic outcomes were more complex that initially hypothesized, it is clear that dopamine, ecdysone, and PDF are all capable of directly acting on AKH cells. Furthermore, our results suggested that these signals interact in a complex, context-specific manner. This study serves as an experimental foundation for further unraveling the mechanisms underlying AKH cell physiology and the endocrine circuits that modulate metabolism and maintain energetic homeostasis.

## 4. Materials and Methods

### 4.1. Fly Husbandry

All flies were maintained in an incubator maintained at 25 °C and under a 12:12 light/dark (LD) cycle unless otherwise stated. Flies were housed in uncrowded conditions and cultured on a standard molasses–malt–cornmeal–agar–yeast medium as described by Bloomington Drosophila Stock Center (Bloomington, IN) [63]. We used the following fly strains: PDF-GAL4 [36] (BL-6869), UAS-tPDF [34] (BL-81110), PDFR-GAL4 [33] (BL-33070), UAS-epac-camps [35] (BL-25407), UAS-mCD8-GFP (BL-5137), UAS-PDFR-RNAi (BL-38347), UAS-DopEcR-RNAi (BL-31981), UAS-Dop2R-RNAi (BL-51423), UAS-D2R-RNAi (BL-50621), UAS-DopR-RNAi (BL-55239), UAS-Rpr (BL-50790), and UAS-GCaMP6s1 (BL-42746).

### 4.2. AKH Cell-Specific Transcriptome

AKH cells expressing GFP under the AKH promoter were microdissected and aspirated into a glass pipette, which was placed in a PCR tube and flash frozen in an ethanol-dry ice bath. The tubes were stored at −80 °C for no longer than three weeks while 10 samples were prepared from 5 fed and 5 starved flies. On the day of RNA amplification, the contents of the PCR tubes were centrifuged, and the RNA from these samples was amplified in parallel using the Arcturus RiboAmp HS PLUS Kit by following the manufacturers protocol (KIT0505, Thermo Fisher Scientific, Waltham, MA, USA). RNA libraries were then prepared using the Kapa Stranded mRNA-Seq library prep kit, and 50 bp single end sequencing was performed on an Illumina HiSeq 4000 at the Duke Center for Genomic and Computational Biology (Durham, NC, USA). These data are available at the NCBI Sequence Read Archive under project number PRJNA642982.

The raw reads were filtered using Trimmomatic v0.36 to remove Illumina adaptors, leading or trailing bases below a quality score of 3, 4-base sliding window average quality below 15, and reads fewer than 36 bp long. Filtered reads were aligned to *Drosophila melanogaster* genome BDGP6.22 using star v2.5 [64], and a count table was generated from coordinate-sorted BAM files using summarizeOverlaps [65]. We identified genes differentially expressed under starvation using the Bioconductor package DESeq2 [66], but no GPCRs were differentially expressed under starvation conditions (adjusted *p* > 0.05).

For single-cell PCR, individual AKH cells expressing GFP were microdissected and stored in Trizol. A single-cell RT-PCR kit from Qiagen was used to make cDNA and amplify using gene specific primers. Methods are described in detail in [7].

### 4.3. Individual Locomotion/Starvation

Three- to five-day-old males were sorted 12–24 h prior to the start of the assay. At ZT0, individual male flies were loaded into 5 × 65 mm polycarbonate plastic tubes capped at one end with a ½-inch piece of yarn. Once loaded, a 200 µL pipette tip filled with standard *Drosophila* media and sealed at one end was placed on the end of the plastic tube. Tubes were then loaded into a Trikentics DAM 2 monitor for 3 days of entrainment on replete media. Total beam counts were monitored continuously with an automated system for the duration of the experiment at 10 min intervals. At ZT0 on the third day, data collection was paused, and media-containing pipette tips were replaced with tips containing a 2% agar water solution. Locomotion was monitored for at least three days or until all flies in starvation had ceased moving for 12 h. Following the experiment, beam breaks were binned into 1 h intervals and used for locomotor analysis. Day 1 was considered a recovery and acclimation period, and the relevant data were removed from analysis. Death was approximated as the timepoint following the last registered beam break, and mean median survival times were calculated as described in [67]. An ANOVA was used to compare these values among genetic manipulations, and a Tukey’s post hoc test was used to determine statistical significance (*p* < 0.05) from control lines.

The numbers of replicates for a given genotype were identical for survival and activity. Replicates used: AKH-GAL4/+ (*n* = 32), AKH > tPDF (*n* = 27), AKH>PDFR RNAi (*n* = 32), AKH>Rpr (*n* = 16), AKH>D2R RNAi (*n* = 26), AKH>DopEcR RNAi (*n* = 32), AKH>Dop2R RNAi (*n* = 31), and AKH>DopR RNAi (*n* = 32). We calculated the mean number of beam breaks per bin for each animal before and during starvation conditions. An ANOVA was used to compare these values among genetic manipulations, and a Tukey’s post hoc test was used to determine statistical significance (*p* < 0.05) from control lines.

### 4.4. Live Cell Imaging

For live cell imaging experiments, corpora cardiaca were dissected and placed in AHL (adult hemolymph-like) [68] solution containing 12 mM trehalose and 3 mM sucrose or 3 mM trehalose and 12 mM sucrose (when stated). Dissections where then placed on a plastic cover slip containing 180 µL of AHL. Explanted AKH cells were then viewed on a Zeiss LSM 710 confocal microscope and visually inspected for damage prior to imaging. All imaging settings were kept constant between experiments. Twenty microliters of treatment was applied in a dropwise manner.

For calcium imaging, a 20 × 0.8 NA objective and a 488 nm laser were used. Z stacks were collected in 10 s intervals. Cells were imaged for 1 min prior to treatment. After imaging, Z stacks were collapsed to maximum-intensity projections. A region of interest was manually drawn for each explanted AKH cell, and total values for pixel intensity were assessed. Values were exported into Excel and normalized to the timepoint immediately prior to application. Dopamine was prepared in 10% PBS AHL solution and added in dropwise fashion to reach the final concentration. A Kruskal–Wallis ANOVA was used for analysis.

For cAMP imaging, a 40 × 0.95 NA objective was used. CFP was excited using a 440 nm laser. Z stacks were collected in 10 s intervals and imaged for 1 min prior to treatment. After imaging, Z stacks were collapsed to maximum-intensity projections. A region of interest was manually drawn for each explanted AKH cell, and total values for pixel intensity were assessed. Values were exported into Excel and adjusted for spillover (SO). Spillover was calculated using CFP-expressing HEK cells under the same conditions previously described and found to be 54%. The FRET ratio was calculated ΔFRET = (YFP − (CFP × SO))/CFP [64]. Data were normalized to the timepoint immediately prior to application, and Friedman’s test was used to determine significance. The concentrations for all hormones used represented those of the least saturating doses and were selected based on pharmacological screens.

### 4.5. Immunostaining

All tissues dissections for immunostaining were conducted in 1× PBS Tx under a standard dissecting microscope. Tissue was then placed immediately into fixative (4% paraformaldehyde, 7% picric acid) for 1 h at room temperature. Tissue was then washed 10× with 1× PBS Tx before blocking with (%) BSA 1× PBS Tx solution for 1 h at room temperature. Next, tissue was placed in 1:1000 αAKH for 1 h at room temperature and then washed ten times before being moved to 1:1000 anti-rabbit Cy3 for 2 h at room temperature. Tissue was then washed and placed into a drop of glycerol to dehydrate it. Finally, the glycerol was removed by a kimiwipe (wicking) and replaced with antifade mounting media. All images were taken on a Zeiss 710 (Zeiss, Germany) confocal microscope.

## Data Availability

The source code for this analysis is available on GitHub [69].

## Supplementary Materials

The following are available online at https://www.mdpi.com/article/10.3390/ijms23084266/s1.
