# Supplementary material for: Modulation of Metabolic Hormone Signaling via a Circadian Hormone and Biogenic Amine in Drosophila melanogaster
Source: Int J Mol Sci. 2022 Apr 12;23(8):4266. doi: 10.3390/ijms23084266 (PMC9030464; doi:10.3390/ijms23084266)

Supplemental Figure S1. Locomotor plots of locomotor activity during replete (black line) and starved (red line) conditions from animals expressing a PDFR-RNAi element (top) or a t-PDF element (bottom) as compared to genetic controls (middle).

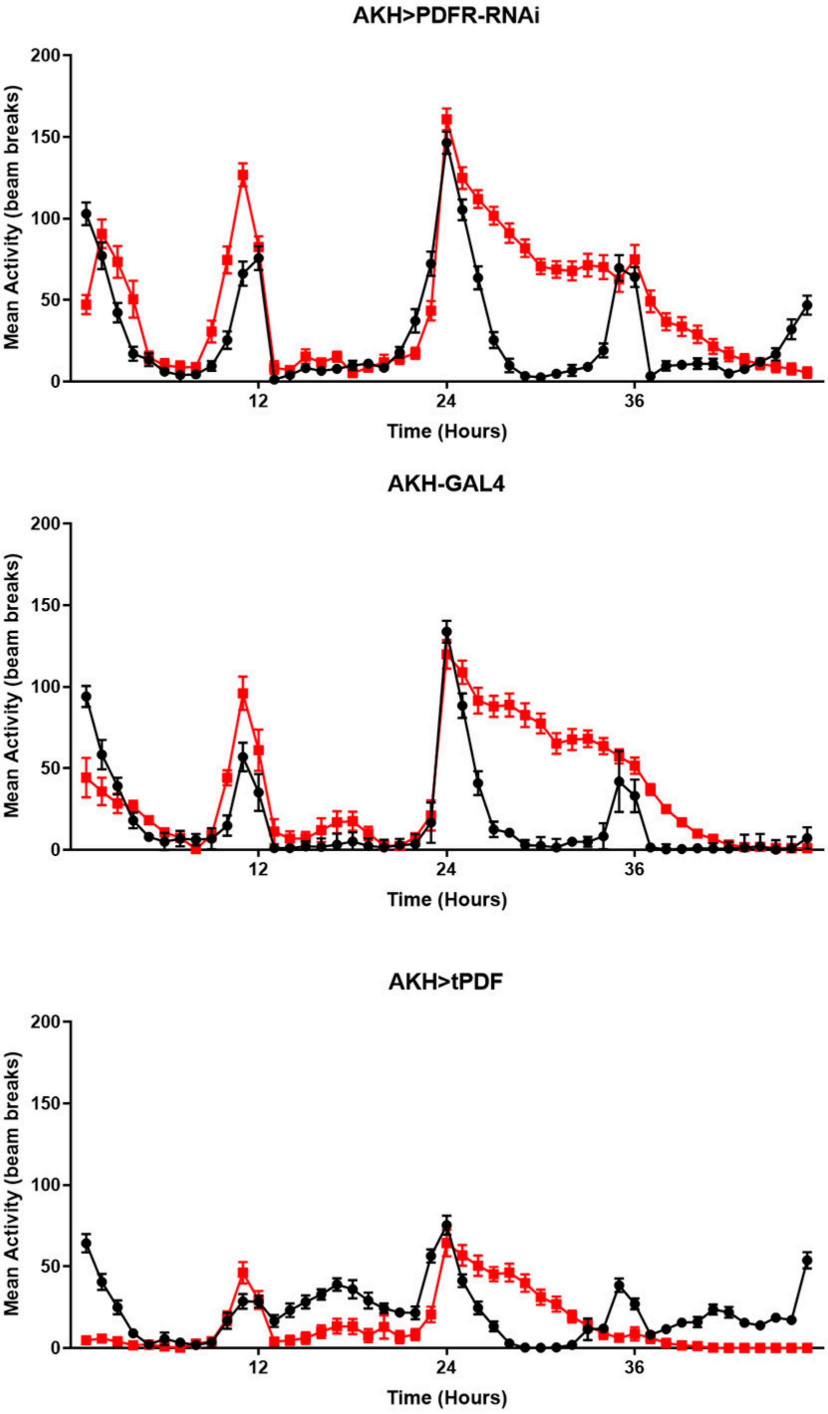

Supplemental Figure S2. Locomotor plots of locomotor activity during replete (black line) and starved (red line) conditions from animals expressing RNAi elements targeting a specified Dopamine receptor as compared to genetic controls.

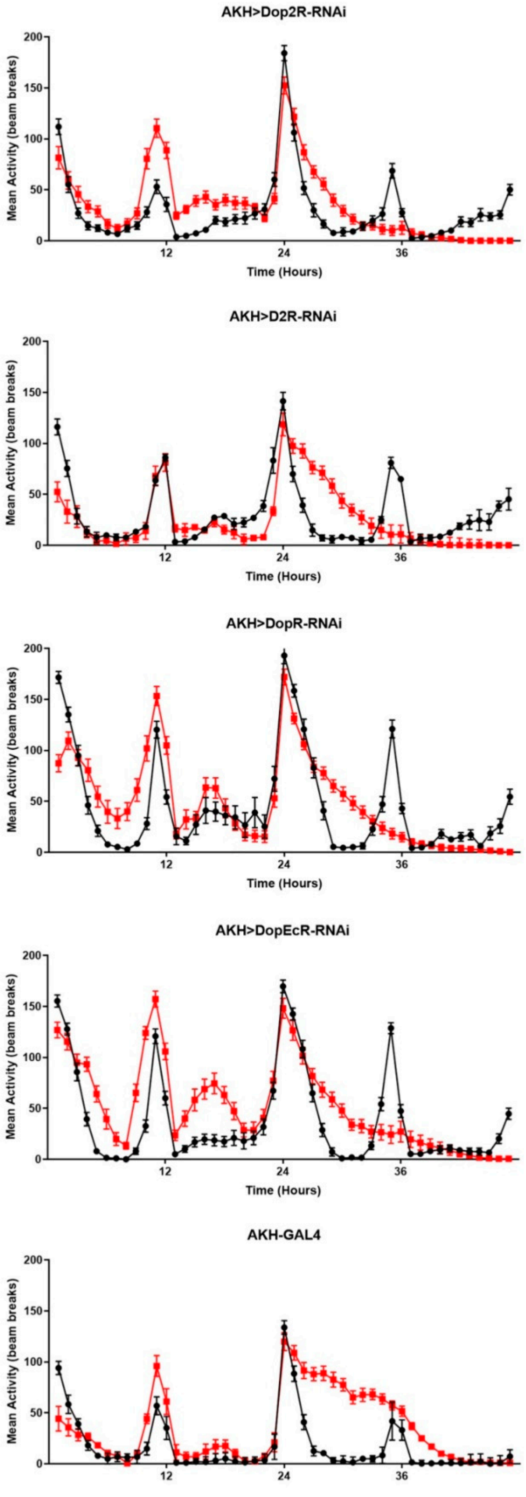

Supplement: Supplementary file 1 [file ijms-23-04266-s001.zip › ijms-1631251-supplementary.pdf]
